# Reversion is most likely under high mutation supply when compensatory mutations do not fully restore fitness costs

**DOI:** 10.1093/g3journal/jkac190

**Published:** 2022-08-03

**Authors:** Pleuni S Pennings, C Brandon Ogbunugafor, Ruth Hershberg

**Affiliations:** Department of Biology, San Francisco State University, San Francisco, CA 94132, USA; Department of Ecology and Evolutionary Biology, Yale University,New Haven, CT 06520, USA; The Santa Fe Institute, Santa Fe, New Mexico, 87501 USA; Rachel & Menachem Mendelovitch Evolutionary Processes of Mutation & Natural Selection Research Laboratory, Department of Genetics and Developmental Biology, The Ruth and Bruce Rappaport, Faculty of Medicine, Technion–Israel Institute of Technology, Haifa 31096, Israel

**Keywords:** reversion, resistance, microbial evolution, compensatory mutation, mutation rate

## Abstract

The dynamics of adaptation, reversion, and compensation have been central topics in microbial evolution, and several studies have attempted to resolve the population genetics underlying how these dynamics occur. However, questions remain regarding how certain features—the evolution of mutators and whether compensatory mutations alleviate costs fully or partially—may influence the evolutionary dynamics of compensation and reversion. In this study, we attempt to explain findings from experimental evolution by utilizing computational and theoretical approaches toward a more refined understanding of how mutation rate and the fitness effects of compensatory mutations influence adaptive dynamics. We find that high mutation rates increase the probability of reversion toward the wild type when compensation is only partial. However, the existence of even a single fully compensatory mutation is associated with a dramatically decreased probability of reversion to the wild type. These findings help to explain specific results from experimental evolution, where compensation was observed in nonmutator strains, but reversion (sometimes with compensation) was observed in mutator strains, indicating that real-world compensatory mutations are often unable to fully alleviate the costs associated with adaptation. Our findings emphasize the potential role of the supply and quality of mutations in crafting the dynamics of adaptation and reversal, with implications for theoretical population genetics and for biomedical contexts like the evolution of antibiotic resistance.

## Introduction

In experimental evolution with microbes, rapid adaptation to new environments often occurs through mutations displaying antagonistic pleiotropy (Cooper and Lenski 2000; MacLean *et al.* 2004; Duffy *et al.* 2006; Avrani *et al.* 2011, 2017; Kvitek and Sherlock 2013). This means that while such mutations are adaptive in the new environment, the fitness improvement comes at the expense of fitness in the original environment (Andersson and Levin 1999; Maisnier-Patin *et al.* 2002; Andersson and Hughes 2010; Conrad *et al.* 2010; Avrani *et al.* 2011, 2017; Melnyk *et al.* 2015). For example, a population of bacteria that has evolved to become resistant to antibiotics while it was in an environment with antibiotics may be less fit than its susceptible ancestor when it returns to its original environment without antibiotics. Thus, when the drug is removed, the mutations conferring resistance are no longer beneficial and, instead, incur a fitness cost. One naive expectation is therefore that when antibiotic usage ceases, the population will evolve “backward” toward being drug susceptible. However, both theory (Dollo’s Law) and data suggest that reversal to ancestral genotypes does not always occur readily (Gould 1970; Bull and Charnov 1985; Teotónio and Rose 2001).

The question of reversal is important for medicine and public health, because attempts to reduce the number of resistant infections by halting the use of antimicrobial drugs have been effective in some contexts (Tang *et al.* 2017; Shen *et al.* 2020), but unsuccessful in others (Andersson and Hughes 2010; Sundqvist *et al.* 2010). For example, the ban on colistin use in animal feed in China in 2017, led to a significant reduction in colistin resistance (from 19% to 5%) in *E*scherichia *coli* sampled from humans, pigs, and the environment (Shen *et al.* 2020). On the other hand, a strong reduction of trimethoprim use in a county in Denmark had a very small effect on trimethoprim resistance rates in *E. coli*, when compared to a control county (Sundqvist *et al.* 2010). In the latter study from Denmark, the authors also measured the cost of resistance in the laboratory and found it to be near zero, which could explain why using no antibiotic had almost no effect on rates of resistance. The varied outcomes of these attempts to reverse drug resistance evolution suggests that the plausibility of reversion is contingent on certain population genetic (or other) contexts.

Even when it is clear that an adaptive mutation comes with a fitness cost, reversion does not always occur. Laboratory studies have examined the persistence or reversion of such costly adaptive mutations by studying bacterial populations that are fixed for mutations that confer resistance to an antibiotic or bacteriophage (e.g. Andersson and Levin 1999; Levin *et al.* 2000; Reynolds 2000; Maisnier-Patin *et al.* 2002; Andersson and Hughes 2010; Avrani and Lindell 2015).

These studies have revealed different reasons why reversion may not always occur. Specifically, many studies have shown that bacteria have a remarkable capability to compensate for the deleterious effects of resistance mutations by acquiring compensatory mutations, which facilitate the maintenance of resistance (Andersson and Levin 1999; Levin *et al.* 2000; Reynolds 2000; Maisnier-Patin *et al.* 2002; Andersson and Hughes 2010; Avrani and Lindell 2015; Debray *et al.* 2022). Other studies have highlighted the role of epistasis in undermining reversal across a fitness landscape (Longzhi *et al.* 2011; Ogbunugafor and Hartl 2016). Many of these findings are built on a foundation of (or are compatible with) theoretical studies that have examined the conditions in which reversibility is possible in light of the effects of beneficial mutations, and the conditions that drive the impact of compensatory mutations (Kimura 1985; Innan and Stephan 2001; Piskol and Stephan 2011). Several theoretical and computational studies have examined this in the setting of microbial evolution (Handel *et al.* 2006; Schulz zur Wiesch *et al.* 2010; Marrec and Bitbol 2020). For example, one such study addressed the role of mutation, migration, and selection in determining the cost of resistance(Jeger *et al.* 2008).

Recent studies have examined a relatively under-studied microbial setting in which the dynamics of compensation and reversal are highly relevant—bacterial adaptation to prolonged resource exhaustion (Avrani *et al.* 2017, 2020; Katz *et al.* 2020). Though it lacks the direct medical relevance of the antibiotic resistance case, this setting is highly relevant for the ecology of bacteria across the biosphere, where resource limitation is the norm. *E. coli* can survive for very long periods of time within spent media (with no supplementation of external nutrients) in a state termed long-term stationary phase (LTSP). We have previously demonstrated that *E. coli* adapts under LTSP through the acquisition of specific mutations within the RNA polymerase core enzyme (RNAPC) (Avrani *et al.* 2017). While these mutations are adaptive under LTSP, they carry a cost that manifests in poorer growth when the bacteria are returned to fresh media.

We have also shown that the tendency of adaptive RNAPC mutations to persist within fresh media was related to the mutation rate of the strain in question (Avrani *et al.* 2020): nonmutator clones recovered through compensatory mutations, while mutator clones (∼110- to 260 times higher mutation rates than the wild type, *WT*) reverted to wild type. Though these specific results were not rigorously explained, the general findings are in line with a literature supporting the role of mutation rate in dictating the dynamics of adaptive evolution in microbes (Shaver *et al.* 2002; Gentile *et al.* 2011; Raynes *et al.* 2011; Wielgoss *et al.* 2013).

In the current study, we use theoretical approaches and computer simulations to gain a better understanding of how mutation rates affect the persistence of costly adaptive mutations and the dynamics of compensation and reversion of such costly adaptive mutations. We consider only stepwise, chromosomal mutation, rather than horizontal gene transfer. In agreement with the long-term stationary phase experimental results (Avrani *et al.* 2020), we demonstrate here that the likelihood of reversion (as opposed to compensation) is low when the mutation supply is low, but it increases with higher mutation rates. However, these results only hold when compensatory mutations do not compensate the fitness cost fully. We find that that reversion is less likely, even at high mutation rates, if there exist compensatory mutations that fully alleviate the costs associated with the adaptive mutations. Thus, the fact that we observe reversion in the data from prior studies (Avrani *et al.* 2020) (where the experiments started with no *WT* individuals present in the population) likely means that perfectly compensating mutations do not exist in this setting.

## Materials and methods

### Note on terminology: “reversion” and “reversal”

In this study, we use “reversal” and “reversion” interchangeably and interpret them as the processes through which an allele reverts back to its ancestral or wild-type form.

Our simulations follow a population after it has adapted to a new environment through the fixation of an adaptive mutation, and the environment has changed back to its original state. The starting point of the simulations is thus a maladapted population, where the adaptive mutation is associated with a cost (*c*). In all simulations shown in the main text, we have used a moderate cost of *c *=* *0.15. We did however, also run our simulations with *c *=* *0.3 and *c *=* *0.05. Changing the cost changes the time scale of events, but not the main outcomes. Results from these other simulations are shown in the Supplementary material. For the remainder of the study, we will refer to the adaptive mutation as a resistance mutation. However, the experimental data we use are from experimental populations adapted to prolonged resource exhaustion (Avrani *et al.* 2020), and we believe that our results are relevant to any adaptive mutation that carries a cost. It is also important to emphasize that our study is focused on the dynamics of adaptation and reversal in the context of point mutation-driven, stepwise evolution, rather than evolution through horizontal gene transfer or plasmid conjugation.

Several types of mutations can occur in our model populations.

1. A reversion mutation removes the resistance mutation and recreates the original *WT* genotype. This mutation occurs with a probability of µ per generation and per bacterial cell. µ is the per-site-per-generation mutation rate of the strain. Bacteria with a reverted genotype are indicated by “*WT*.”
2. Compensatory mutations reduce the cost of the resistance mutation by *p* such that the cost becomes c×(1−p). *p* is set to 0.5 in most of the simulations, which means that a compensatory mutation compensates for half of the cost of the resistance mutation, later we vary the value of *p* to determine how it changes outcomes. We allow *n* different compensatory mutations to happen, where *n* is set to 100 for most simulations; later we vary the value of *n* to determine how it changes outcomes. Each of the *n* compensatory mutations occurs with a probability of µ per generation and per bacterial cell. Bacteria with a compensatory mutation are indicated by “Com” in the figures. In our simulations, bacteria can only acquire 1 compensatory mutation.
3. Bacteria that carry a compensatory mutation can acquire a subsequent reversal mutation in addition to their compensatory mutation, with a probability of µ per generation and per bacterial cell (though this feature is turned off for some of the simulations). Bacteria that carry a compensatory mutation and are then reverted to *WT* are indicated by “compensated reversal” or “*CR*” in the figures. The fitness of the *CR* strains is set to be equal to *WT* fitness.

### Additional notes on simulations and associated calculations

We carried out forward-in-time (Wright–Fisher) computer simulations using a custom R script. In the simulations, a population of size *N* (10,000) individual bacteria is followed over G (100 or 500) generations. The per-site-per-generation mutation rate, µ, varies between $10^{-3}$ and $10^{-7}$. The per-site-per-generation mutation supply rate (Nμ) therefore varies between 0.001 and 10.

While some of the mutation rates we used are significantly higher than what is observed in the experimental *E. coli* populations, they lead, in combination with the chosen value for *N*, to a range of mutation supply rates that are realistic and that lead to results similar to what we observed in experiments.

Each new generation is generated by multinomial sampling to include differences in fitness and the stochastic nature of reproduction. In addition to the simulations, we used a simplified mathematical approximation to calculate the probability of different outcomes (see Supplementary material). All code is available at: https://github.com/pleunipennings/CompensatoryEvolution.

## Results

### Possible evolutionary outcomes

**Fig. 1.**
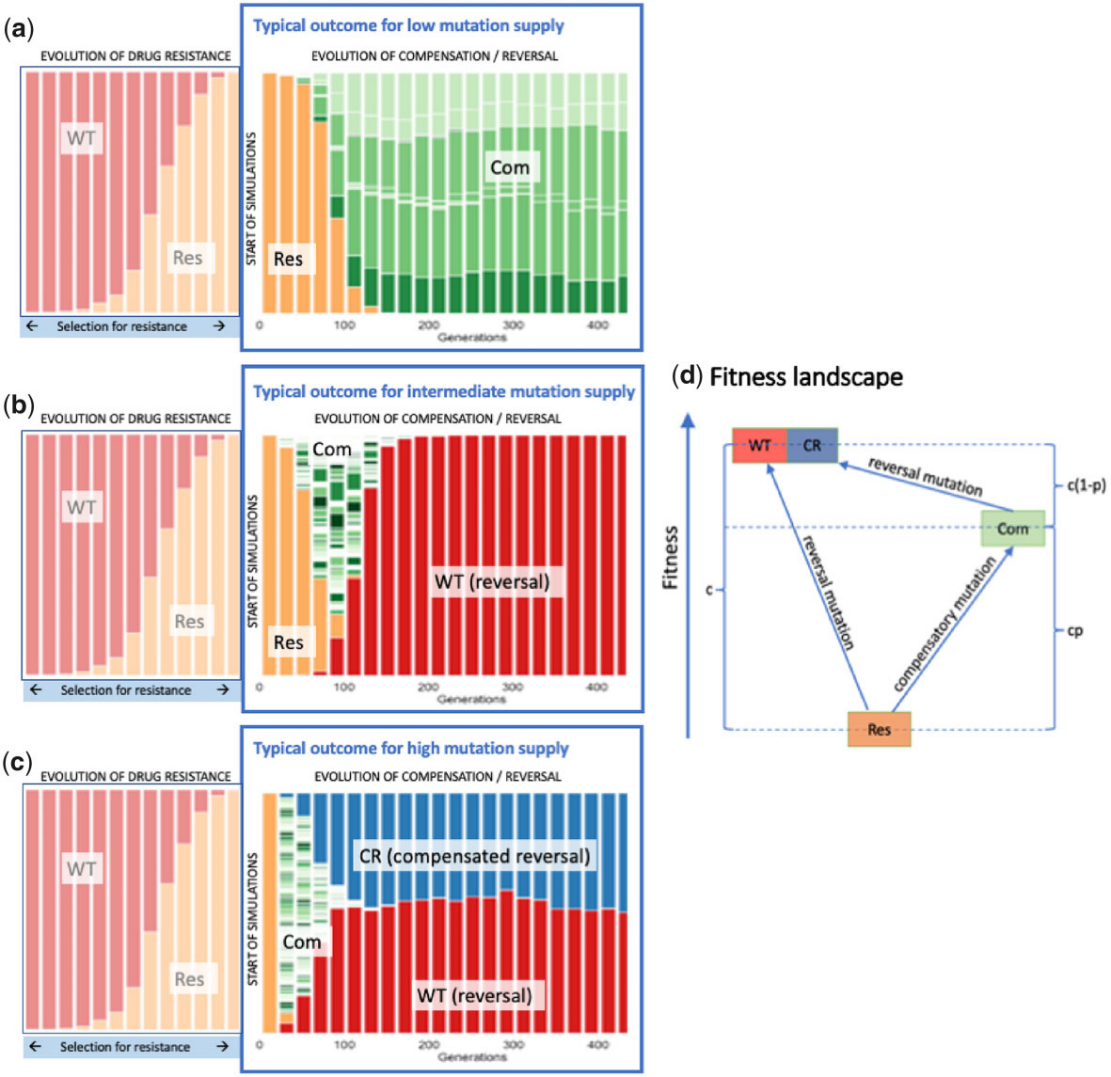
Illustrative simulations with different mutation supply rates. Each panel shows the result of a single simulation. For illustration purposes, we include for all 3 scenarios the time where the (orange) resistant allele rises in frequency due to strong selection until it is fixed in the population. This first part of the graph is not based on a stochastic simulation, but uses a deterministic model of selection on a single resistant allele. This study focuses on what happens after the resistant allele is fixed. a) A low mutation supply (population-wide per-generation mutation rate) typically leads to the fixation of compensatory mutations in the first 500 generations. b) An intermediate mutation supply usually leads to reversal mutations (leading to *WT* genotype) outcompeting the compensatory mutations. c) A high mutation supply rate typically leads to *WT* reversal mutations occurring both by itself (red “WT”), and on the background of a compensatory mutation (blue, “*CR*”). The *X*-axis represents generations, and the *Y*-axis corresponds to the frequency of a given genotype in the population. Because there are multiple possible compensatory mutations, we have chosen to depict them with different shades of green. Here, all compensatory mutations have the same compensatory effect. d) An illustration of the fitness landscape. Red = *WT*, orange = resistant, blue = compensated and reverted.

We started by carrying out simulations with varying parameters to determine their possible outcomes. We identified 3 main possible outcomes, described in Fig. 1. For illustration purposes, we show for all 3 scenarios a resistant allele (orange, “Res”) that rises in frequency due to strong selection until it is fixed in the population. This study focuses on what happens after the resistant allele is fixed, and when there is no longer selection for resistance. Will the population evolve to wild type? Or will the cost of resistance be compensated instead?

In a first possible scenario (Fig. 1a), which occurs when the mutational supply in the population is low (around 1 reversal mutation every 100 generations, Nμ≤0.01), compensatory mutations (of which there are *n *=* *100) arise and fix in the population before a successful *WT* reversal mutation occurs.

A second possible scenario (Fig. 1b) typically occurs when there is an intermediate mutation supply in the population (approximately 1 *WT* reversal mutation every 10 generations, Nμ=0.1). Here, several compensatory mutations likely arise in the first generation (green). However, none of the compensatory mutations can fully restore fitness, and in this higher mutation rate scenario, a *WT* reversal mutation (red) occurs before the compensatory mutations fix in the population. The *WT* reversal mutation has higher fitness than the compensatory mutations and once established will fix in the population.

A final scenario (Fig. 1c) is likely with a high mutation supply rate (1 or more *WT* reversal mutations every generation, Nμ≥1). Here, a different pattern emerges: both compensation (green) and *WT* reversal (red) arise (as in Fig. 1b); however, before the *WT* reversal mutations fix in the population, the bacteria with compensatory mutations also acquire *WT* reversal mutations. Now there are *WT* reversal mutations on 2 different genetic backgrounds: with and without a compensatory mutation. This scenario resembles a “soft sweep” where the reversal mutation occurs multiple times, both on 1 or more compensated genotypes (red) and on 1 or more uncompensated genotypes (red) (Hermisson and Pennings 2017). Because these 2 *WT* reversal types have equal fitness in our model (and likely often in reality), compensated and uncompensated alleles can coexist at intermediate frequency for a long time.

In the next section, we will determine how often each of the 3 scenarios happens given a set of population genetics parameters.

### Direct *WT* (genotype) reversal is more likely when Nμ is higher, the mutational target for compensatory mutations is lower and when compensation is less effective

First, we sought to determine the probability of the scenario outlined in Fig. 1b (where a *WT* reversion mutation outcompetes compensatory mutations) vs the scenario in Fig. 1a (compensatory mutations fix in the population). For simplicity, we do not allow *WT* reversion mutations to occur on an already compensated genotype (that is scenario 1C is not possible).

Reversion mutations toward the *WT* genotype have higher fitness than the allele carrying compensatory mutations. Therefore, if a *WT* ancestor reversal mutation occurs and escapes genetic drift, it will outcompete the compensatory mutations. However, if the mutation supply rate in the population (Nμ) is low, it is possible and likely that the compensatory mutations fix before a successful *WT* reversal mutation occurs. We can see this situation as a race between fixation of the (very common) compensatory mutations and occurrence of a (rare) *WT* reversal mutation and there are only 2 possible long-term outcomes: either the entire bacterial population will consist of those with compensatory mutations or with the reverted *WT* genotype.

**Fig. 2.**
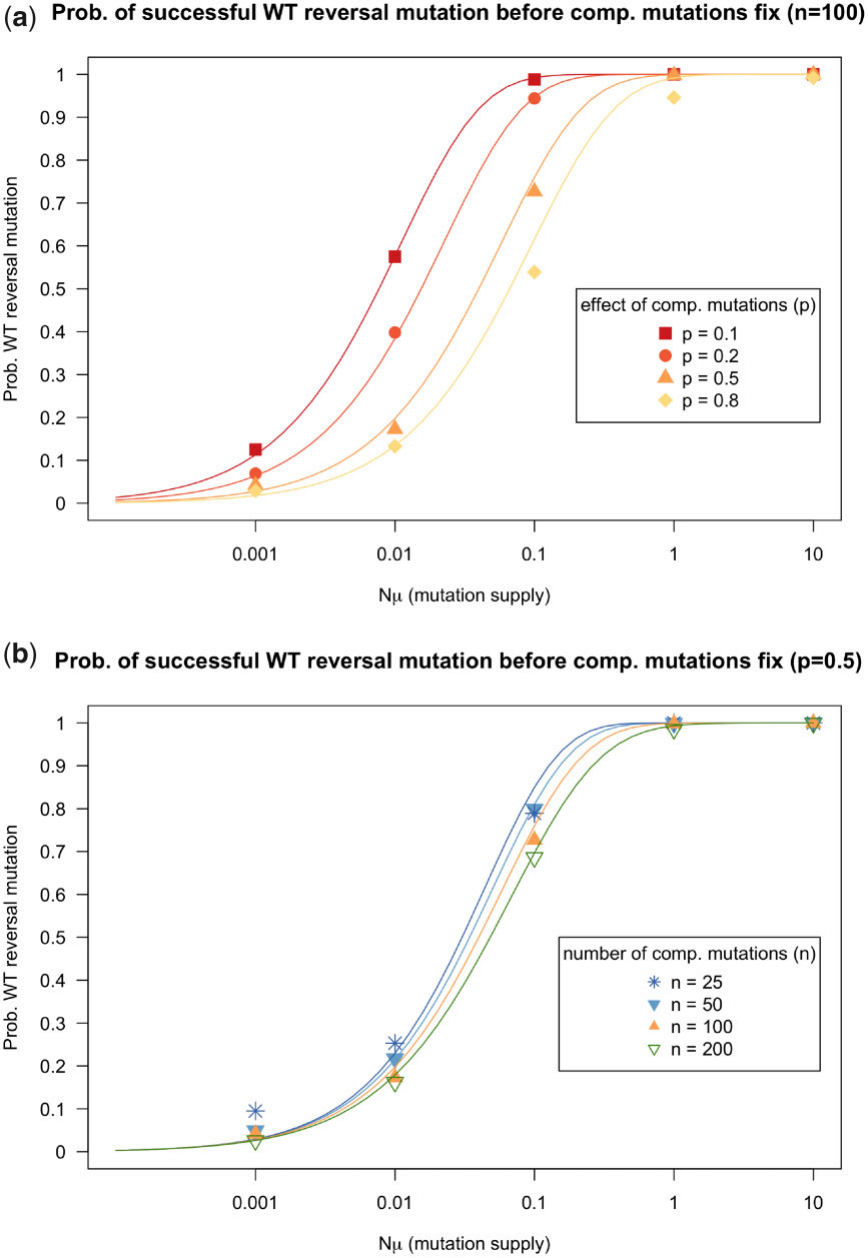
a) The probability that a *WT* reversion mutation occurs before the compensatory mutations (Com) fix in the population. In these simulations, clones with compensatory mutations cannot acquire a second mutation (i.e. they cannot revert to *WT*). Simulations are run for different values of *μ*, the mutation rate, and different values of *p*, the compensatory effect. Simulations are run for 500 generations and for each parameter combination we run 1,000 simulations. Lines are based on the mathematical model. b) Same as (a), but simulations are run for different values of *n*, the target size for compensatory mutations. Simulations are run for 500 generations. Other parameters: population size *N *=* *10,000, cost *c *=* *0.15, effect of compensatory mutations *P = *0.5. The *WT* is considered to be present (and expected to fix) if at least present in 10 individuals at the end of the simulation (0.1% of the population).

We find that *WT* genotype reversal mutations are most likely to occur before the compensatory mutations fix when the mutation supply is high (shorter wait time for a *WT* reversal mutation, see Fig. 2a), when the mutational target size for compensatory mutations (*n*) is low (Fig. 2b) or when the effect of compensation (*p*) is small (Fig. 2a). The effect of small values for the *n* and *p* parameters makes the fixation of the compensatory mutations slower, which gives more opportunity for the *WT* genotype reversal mutations to occur. The figures show both simulations and calculations from the simplified mathematical model (see Fig. 2).

Note that for high values of Nμ our analytical calculations somewhat overestimate the probability a successful *WT* mutation occurs (the lines are higher than the points in Fig. 2, a and b for high Nμ values). This could be because we may, in the analytical calculations, overestimate the fixation probability (*P*_fix_) for mutations when there are other mutations sweeping at the same time. The analytical model assumes that the mutation is the only mutation that is increasing in frequency and that the fixation probability is determined by the selection coefficient in the generation that the mutation occurs. In reality, there are likely other mutations increasing in frequency at the same time and thus the population fitness is increasing faster than assumed lowering the fixation probability.

### Evolution of the *WT* in the context of compensatory mutation occurs when the mutation supply is high

**Fig. 3.**
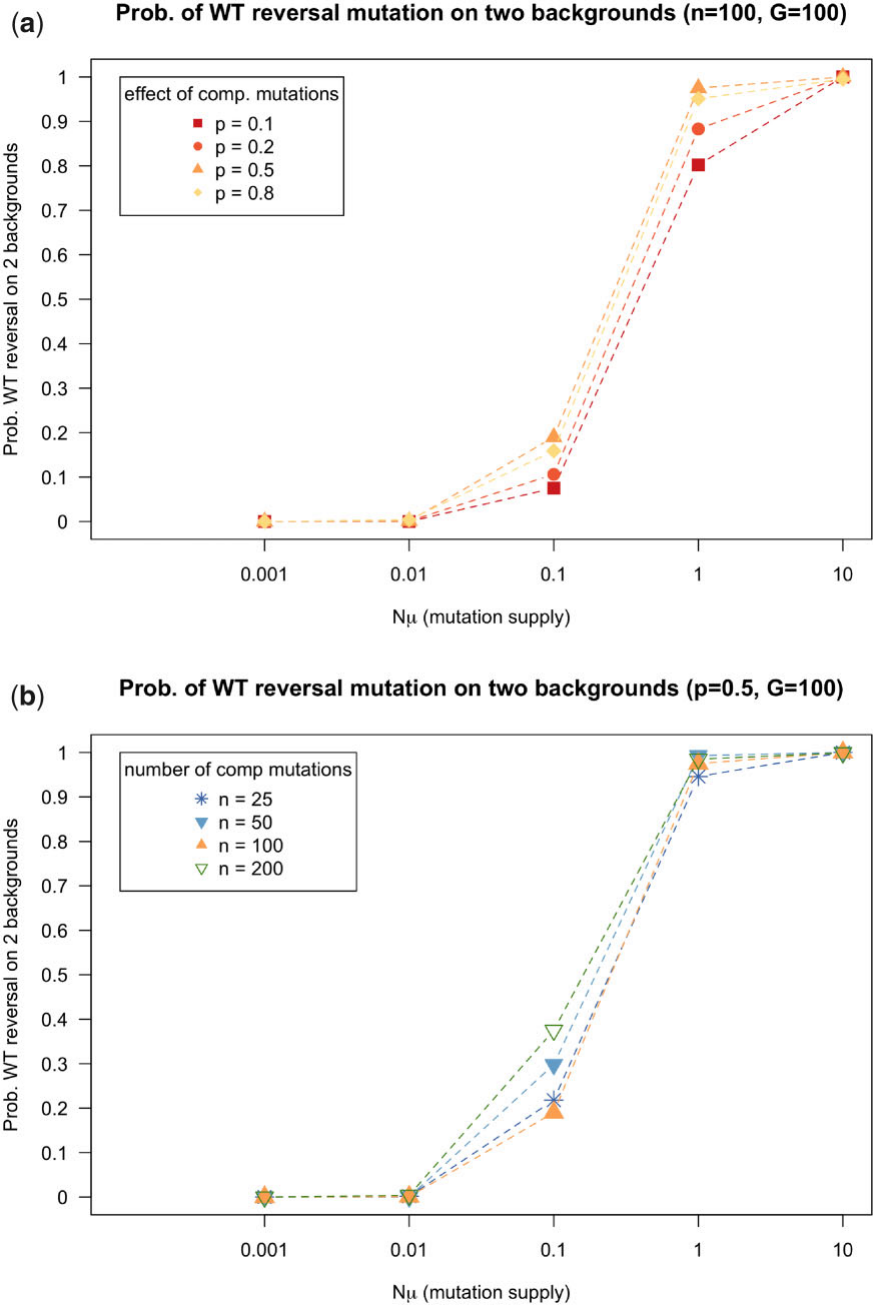
a) The probability that a reversal mutation to the *WT* phenotype occurs on 2 different backgrounds, i.e. from the resistant clone without a compensatory mutation (Res to *WT*), and a resistant clone carrying a compensatory mutation (Com to *CR*). In these simulations, resistant clones with compensatory mutations can acquire a second mutation (i.e. they can revert to the wild-type phenotype via compensated resistance, *CR*). Simulations are run for 100 generations and for each parameter combination we run 1,000 simulations. b) Similar to (a), but simulations are run for different values of *n*, the target size for compensatory mutations. Simulations are run for 100 generations. Other parameters: *N *=* *10,000, *c *=* *0.15, *n *=* *100, *P = *0.5. A mutation is considered to be present if at least present in 10 individuals (0.1% of the population).

Here, we determined the probability that scenario 3 (Fig. 1c) occurred, with reversal to a wild-type phenotype via multiple routes: resistance reversal (to the *WT* allele) and compensated reversal (to the *CR* allele). When the mutation supply is high (Nμ≥1), the wild-type phenotype reversal mutation can occur on 2 different backgrounds. It can occur by itself (indicated by the red color in Fig. 1) or on a background that already carries a compensatory mutation (indicated by the blue color and letters “*CR*” in Fig. 1). We show here that there is a fairly sharp transition between 2 mutation supply regimes. If Nμ is 0.1 or lower, it is uncommon to see the wild-type phenotype reversal mutation on 2 backgrounds in the simulations. However, if Nμ is 1 or higher, we see it in nearly all simulations (see Fig. 3). The effects of *p* (effect of compensatory mutations), *n* (number of compensatory mutations), and *G* (number of generations) are quite small (results for *G *=* *500 shown in the Supplementary material).

### What to expect in an experimental setting?

**Fig. 4.**
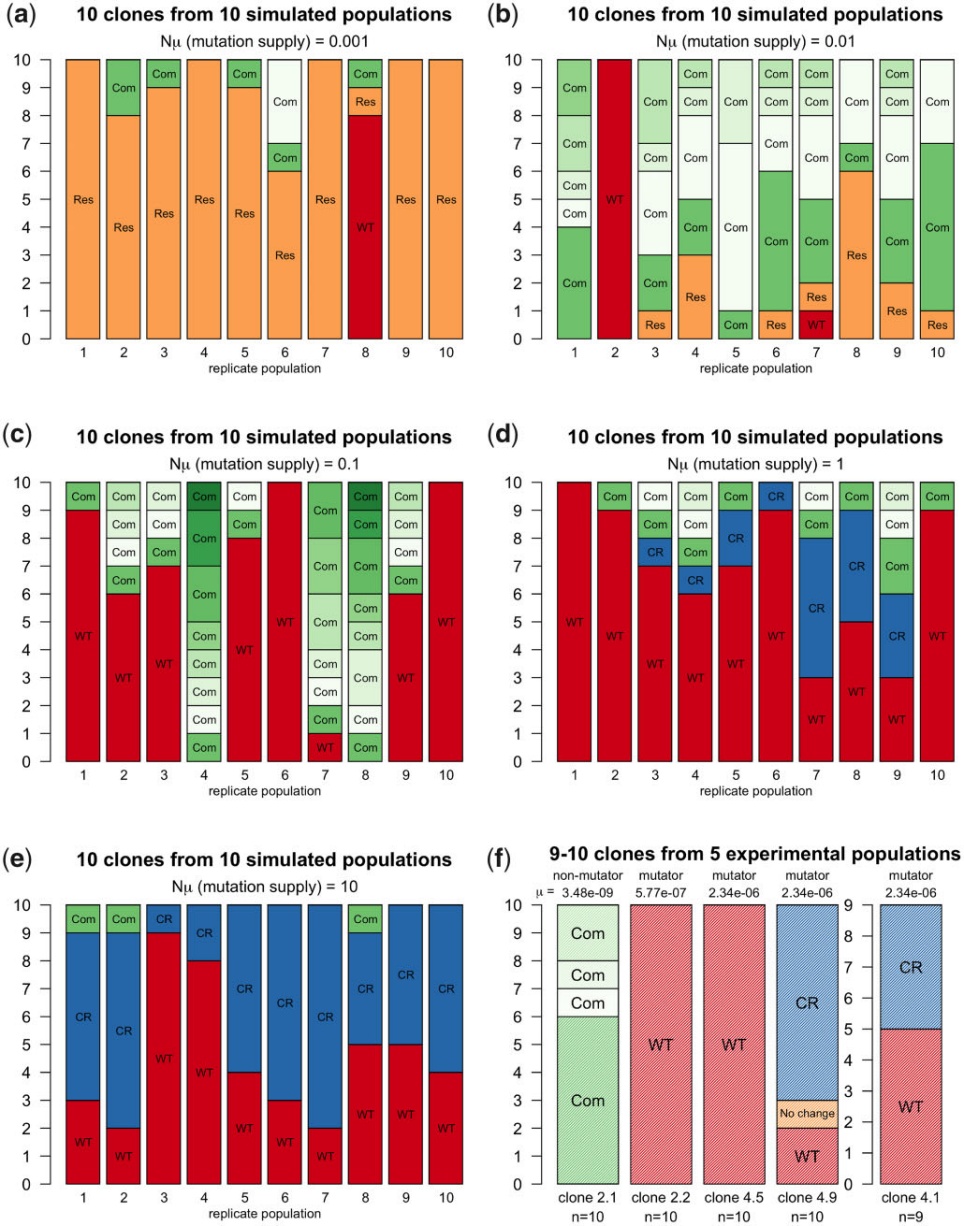
The dynamics of resistance, compensation, and reversion in computational experiments (a–e) and previously published experimental results (f). *Res* = resistant alleles; *Com* = compensated; *WT* = wild type; *CR* = compensated reversal. Different shades of green correspond to different compensatory mutations. Note that in (f) (the experimental results), the nonmutator yielded 4 types of compensatory mutations and no reversions, while the mutators yielded only reversions to the wild type, or combinations of reversions and compensatory mutations within the same population. There was 1 sample from clone 4.9 that did not have a reversal nor a compensatory mutation (indicated in orange). These results from the mutator clones are similar to what is observed in the computational experiments, when Nμ was set to 1 or 10, in (d) and (e). Other parameters: *N *=* *10,000, *c *=* *0.15, *P = *0.5, *n *=* *100.

When conducting computer simulations, 1 can easily run a script hundreds or thousands of times and then determine the precise genetic makeup of the simulated population. This is what we did, the results described in Figs. 2 and 3. However, when studying the evolution of bacterial populations in the laboratory, we are often limited to a smaller number of populations and we may only be able to sequence a small number of individuals from these populations. Therefore, to get a better sense of what to expect in an experimental setting, we ran simulations for just 10 populations for 100 generations and simulated sampling 10 bacterial cells from those populations. Figure 4, a–e depicts the outcomes of 10 independent simulation runs for each of 5 different values of Nμ. We find very different outcomes, depending on the Nμ value (mutational supply in the population). Outcomes vary between replicate runs as well. Still, it is clear that some outcomes are more common for some values of Nμ.

For these simulations, all possible compensatory mutations have the same effect size and compensate half of the cost of the resistance mutation (comp = 0.5), so that the relative fitness values are 0.85 (for the resistant genotype), 0.925 (for the compensated genotype), and 1.0 (for the reverted *WT* genotype).

When Nμ equals 0.001 (Fig. 4a), the populations do not evolve much over 100 generations and most of the sampled bacteria still carry the costly resistance mutation (indicated by the color orange and the word “*Res*”), with some compensatory mutations in some of the populations too (indicated by the color orange and the word “*Com*”). When Nμ equals 0.01 (Fig. 4b), it is more likely that compensatory mutations reach high frequencies (green colors). In a few cases, the *WT* reversal mutation reaches a high frequency too (indicated by the color red and the word “*WT*”). When Nμ equals 0.1 (Fig. 4c), we observe reversal to *WT* and compensatory mutations but do not yet see reversion combined with compensation (indicated by the color blue and the word “*CR*”). This is because in this regime, the mutation rate is not high enough for double mutants (those conferring compensation and reversal). When Nμ is 1.0 (Fig. 4d), reverted clones become much more frequent than compensated clones and we begin seeing *CR* clones as well. When the mutation supply is even higher (Nμ is 10, Fig. 4e), the most common outcome, by far, becomes reversion (either by itself, in red, or in addition to a compensatory mutation, in blue), leading to the loss of the costly adaptation.

In summary, we find that a 10- or 100-fold increase in mutation rates is sufficient to greatly modify probability of reversing the evolution of costly adaptive alleles. With lower-mutation rates we see less reversion (with and without compensation) and with higher mutation rates we see more reversion (with and without compensation). When Nμ is 1 or 10, we see the *WT* reversal mutations on different backgrounds.

### Comparison between computational results and previous experimental results

The results of our simulations correspond well with the experimental results that were previously observed in a study of costly adaptations in the RNA polymerase core enzyme in experimental *E. coli* long-term stationary phase populations (Avrani *et al.* 2020). In these experiments, 5 clones with the previously adaptive mutation were used to initiate ∼100-generation serial dilution experiments, to see if they would lose the adaptive mutation or not. Reversal or compensation was expected because the adaptive mutation was shown to carry a cost to growth within fresh rich media.

One of the 5 clones used in this experiment had a mutation rate that is normal for *E. coli* (clone 2.1 nonmutator). The other 4 clones had a mutation in a mismatch repair gene leading to a substantially higher mutation rate (clones 2.2, 4.1, 4.5, and 4.9 mutator strains) (Katz *et al.* 2020).

Results for the nonmutator clone (2.1) in the experiment (Fig. 4f) correspond well to the simulations where Nμ = 0.01 or 0.1 (Fig. 4, b and c). In this experiment, 4 different compensatory mutations (in green) are observed in the population sample, but no reversal is observed. This observation is important because it shows that there exist different possible compensatory mutations, which together have a mutation supply rate that is higher than the reversal mutation.

On the other hand, results for the 4 mutator clones were very different (Fig. 4f). For 3 of the 4 mutator clones, all sequenced bacteria (9 or 10 per clone) had reverted to *WT*, whereas for one of the mutator clones (clone 4.9), 9 of 10 sequenced bacteria had reverted. This means that in most cases, the costly RNAPC adaptation was lost. These results are similar to what is observed in the computational experiments outlined in Fig. 4, d and e, when Nμ was set to 1 or 10.

The results of our simulations are therefore consistent with previously published experimental results: we observe a shift from observing the persistence of costly adaptations through compensation, to the loss of costly adaptations due to reversion, for ∼100-fold changes in the rate of mutation.

### Evolutionary dynamics change when fully compensating mutations are included

In the simulations described above, we assumed that compensatory mutations do not fully alleviate costs associated with costly adaptations (*p *<* *1). Next, we asked whether we would find similar results if 1 or a few of the compensatory mutations would fully restore fitness.

We do this by changing the fitness effect of 1, 3, 5, or 10 specific compensatory mutations such that strains with these mutations will have a fitness equal to the wild type (1), whereas strains with the other compensatory mutations have fitness 1−c(1−p). We find that including perfectly compensating (*p *=* *1) mutations in our simulations changes the outcomes drastically.

**Fig. 5.**
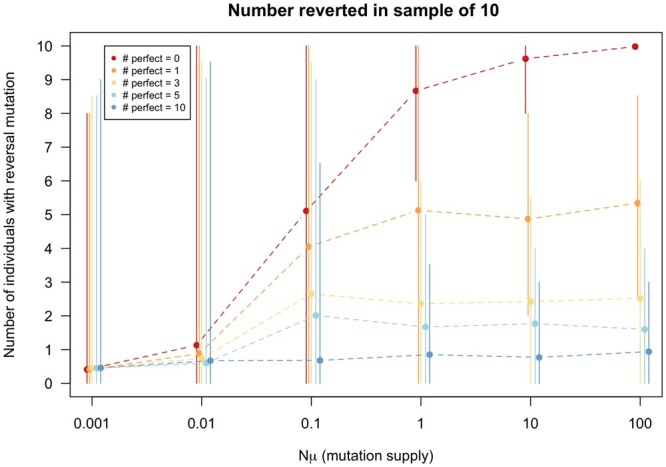
Simulations of evolution across mutation rates under different numbers of available fully compensatory mutations. The *x*-axis corresponds to ranges of mutation supply (Nμ), and the *y*-axis shows the number of sampled bacteria where reversion to the wild type occurred. Here, we observe that even the presence of a single fully compensatory mutation (dark orange line) greatly decreases the likelihood that the wild-type allele will arise. The simulations were run for 100 generations. Other parameters: N=10,000,c=0.15,p=0.5,n=100. The error bars represent the 95% confidence interval (from the 2.5 percentile to the 97.5 percentile of the simulation runs).

In Fig. 5, we show that when there are no perfectly compensating mutations, the fraction of the sample that carries the reversal mutation goes up with increasing mutation supply rate (Nμ). This can also be seen in Fig. 4, a–f. With higher mutation rates, we see more and more reversals. However, this effect of increasing mutation rates is much weaker when there are perfectly compensating mutations.

Including even just 1 fully compensating mutation reduces the average fraction of the sample that carries the reversal mutation when the mutation supply is high. Specifically, when the mutation supply is 10 or 100, high enough that mutations are virtually guaranteed to occur immediately, 1 perfectly compensating mutation (with the same mutation rate as the reversal mutation) will make that, on average, half of the population will carry the compensatory mutation at the end of the experiment, the other half being wild type. While somewhat counterintuitive, this can be explained as follows. In this case, there are 2 mutations that have the same mutation rate and the same, high, beneficial effect—we expect both of them to occur rapidly and increase in frequency. At fixation, each is expected to have reached 50% frequency. When there are more than 1 perfectly compensating mutations, the expected fraction of the population that carries the reversal mutation decreases to $\frac{1}{m+1}$ where *m* is the number of perfectly compensating mutations. The results in Fig. 5 are consistent with this expectation. When there are 3 perfectly compensating mutations, the number of reverted clones in a sample of 10 is expected to be 2.5.

Note that in the experimental results (Fig. 4F), we observe that in the mutator populations, the reversion mutation is seen in nearly all sequenced bacteria, which is compatible with a model where there are no perfectly compensating mutations available to the evolving populations. The experimental outcome would be unlikely if there were any perfectly compensating mutations available.

In this manner, the simulation results outlined in Fig. 5 help to diagnose molecular evolutionary specifics of the empirical findings depicted in Fig. 4f. They suggest that, in the experimental populations, there were no perfectly compensating mutations available. Even if only a single fully compensating mutation was available, we would have expected to see that only half (on average) of the population would have carried the reversal mutation and the other half would have carried a compensatory mutation only.

If compensatory mutations tend to only partially alleviate costs associated with adaptations, it is reasonable to expect that reversion mutations may ultimately occur and rise in frequency, even in clones that have already acquired a compensatory mutation. In our simulations of populations with higher mutation rates, we often observe clones that carry both a compensatory mutation and a reversion mutation (indicated in blue and with the letters “*CR*”). This suggests that reversion after partial compensation would also occur in lower-mutation-rate populations if we would follow the populations for (many) more generations.

## Discussion

This study used population genetic theory and computational methods to examine the evolutionary dynamics of reversion and compensation in a context where mutations that confer adaptation to 1 setting confer a cost in another. Specifically, it sought to examine the conditions favoring reversion or compensation with regard to (1) differing mutation rates and (2) different values for the magnitude of fitness recovery for compensatory mutations. Importantly, this examination applies very directly to the results of experiments in *E. coli*.

Consistent with previously published laboratory findings, we find that several aspects of the evolutionary dynamics of resistance and compensation differ significantly at higher mutation rates (Avrani *et al.* 2020). At low mutation supply rates, the predominant genotypes are those that have remained resistant, either with or without compensatory mutations. In higher mutation rate settings, reversion (to the wild type) and compensated reversal (genotypes where both compensatory mutations and reversal mutations arise) are the predominant outcomes. It is important to note that, in these settings, all resistance mutations have the same cost, and no compensatory mutations are fully restorative, which explains why reversion to the wild type can occur readily in high mutation rate contexts even after compensation. However, in settings where there are compensatory mutations that can fully restore fitness to wild-type values (perfect compensation) in the original environment, reversion occurs much less readily. This effect is especially visible in high mutation settings (see Fig. 5).

Our results are limited by the boundaries of our questions, and by extension, our modeling approach: we assume a simple fitness landscape with no sign epistasis between mutations. Resistance in our model is caused by a single mutation, which is important to consider because multistep reversion can be limited on rugged, complex fitness landscapes defined by sign epistasis (Longzhi *et al.* 2011; Draghi and Plotkin 2013; Chou *et al.* 2014; Ogbunugafor and Hartl 2016). This study also focuses on the case of a single environmental shift, from an environment where a resistance mutation is beneficial to an environment where it is detrimental. Alternatively, a growing literature examines how different (or dynamic) environments may affect mutation effects and evolutionary dynamics (Bjorkman *et al.* 2000; Mustonen and Lässig 2009; Tanaka and Valckenborgh 2011; Cvijović *et al.* 2015; Ogbunugafor 2022).

Finally, we use a Wright–Fisher model with discrete generations and an effective population size of *N* = 10,000. While these sorts of models are standard, we should note 2 caveats: (1) we do not know the effective population size in the experimental populations and (2) the demographics of the experimental populations are certainly more complex than exist in the Wright–Fisher model that we use. While do not believe that these features are likely to affect our results qualitatively, they are important questions for future exploration.

## Conclusion

This study highlights the utility of theoretical and computational approaches to interpret findings from experimental evolution of resistance, compensation, and reversion. They fortify notions that mutation rate is a strong modulator of microbial evolution (Maciá *et al.* 2005; Sprouffske *et al.* 2018; Katz *et al.* 2020). Given this, the resistance management paradigm should continue to consider the effect varying mutation rates can have on the tendency of costly adaptations to persist or revert. Specifically, when reversal does not happen as expected, there could be several reasons for this including low mutation supply rates and the existence of perfectly compensating mutations (Andersson and Levin 1999; Levin *et al.* 2000; Reynolds 2000; Maisnier-Patin *et al.* 2002; Andersson and Hughes 2010; Avrani and Lindell 2015).

These findings transcend biomedical settings and are relevant to other contexts where compensatory mutations play a role. For example, recent studies have identified that the fitness effects of compensatory mutations are often partial in experimental evolution for cellular modules (Venkataram *et al.* 2020), which supports the importance of our results for how complex phenotypes may evolve. More broadly, future examinations of experimental evolution should carefully consider the mutation rate background in which evolutionary dynamics occur and examine the population genetic (and mechanistic, if possible) underpinnings of compensation. These features can play an underappreciated role in the pace and direction of adaptive evolution, with implications beyond the evolution of drug resistance or even the microbial world.

## Data availability

The experimental results discussed in the article can be found in the original reference (Avrani *et al.* 2020). All code is available on github: https://github.com/pleunipennings/CompensatoryEvolution.

Supplemental material is available at *G3* online.

## Supplementary Material

jkac190_Supplementary_Data
