# Supplementary material for: Reversion is most likely under high mutation supply when compensatory mutations do not fully restore fitness costs
Source: G3 (Bethesda). 2022 Aug 3;12(9):jkac190. doi: 10.1093/g3journal/jkac190 (PMC9434179; doi:10.1093/g3journal/jkac190)
Supplement: jkac190_Supplementary_Data [file jkac190_supplementary_data.pdf]

Mathematical supplement

Probability that the wild type arises before compensatory mutation fixes

We calculate the probability that the WT mutation occurs before fixation of the compensatory mutations using the following assumptions. Bacteria can either acquire a compensatory mutation or a WT reversal mutation, not both.

The absolute fitness of resistant bacteria $w_{res} = 1 - c$. The absolute fitness of the compensatory mutant $w_{comp} = 1 - c(1 - p)$, see Figure 1D.

We assume that compensatory mutations increase in frequency deterministically, starting from 1 individual due to selection and mutation, and the number of resistant bacteria decreases deterministically, starting from $N - 1$.

The population size of the compensatory bacteria follows:

$$N_{comp}[t+1] = N_{comp}[t] \cdot w_{comp}/\bar{w}[t] + N_{mut}$$

where $\bar{w}$ is the average population fitness. The population size of resistant bacteria follows:

$$N_{res}[t+1] = N_{res}[t] \cdot w_{res}/\bar{w}[t] - N_{mut}.$$

The number of mutants from resistant to compensated is

$$N_{mut} = (1 - (1 - \mu)^n) \cdot N_{res}[t] \cdot w_{res}/\bar{w}[t]$$

per generation.

Now, for each generation, we have the number of resistant and compensated individuals and the average population fitness, which allows us to calculate selection coefficient and thus fixation probability for a WT reversal mutation.

In a given generation, t, the selection coefficient of a WT reversal mutation is $s_{wt}[t] = w_{wt}/\bar{w}[t] - 1$ (where $w_{wt} = 1$). The fixation probability of such mutation is

$$P_{fix}[t] = (1 - e^{(-2s_{wt}[t])})/(1 - e^{(-4Ns_{wt}[t])})$$

(Kimura 1957; Patwa and Wahl 2008), which simplifies to $P_{fix}[t] = 2s_{wt}[t]$ when s is not too large (Haldane 1927).

The probability that at least one successful reversal mutation occurs in a generation, $P_{wt}[t]$, is calculated as 1 minus the probability that none of the resistant individuals mutates and fixes.

$$P_{wt}[t] = 1 - (1 - \mu \cdot P_{fix}[t])^{N_{res}[t]}$$

The total probability of a successful WT reversal mutation before the compensatory mutations fix is calculated as a sum over all generations:

$$P_{tot} = \sum_{t=1}^{\infty}(P_{wt}[t]) \cdot \prod_{d=1}^{t-1}(1 - P_{wt}[d])$$

where the product denotes the probability that no successful WT mutation had occurred in previous generations.

Even though we sum to infinity, the opportunity for WT mutants in this model only lasts until the compensatory mutations have fixed. Over time, as the number of resistant (non-compensated) bacteria goes down, the mutational supply of WT reversals goes down too (because here only resistant bacteria can mutate to WT). In addition, the population fitness ($\bar{w}$) goes up, so the selection coefficient (s_{wt}) and therefore the fixation probability P_{fix} for WT goes down. To calculate the probabilities for Figures 2A and 2B we sum over 500 generations.

Experimental supplement

For further clarity on the experimental results, we will briefly describe the results from 5 of the experimental *E. coli* populations as observed in (Avrani *et al.* 2020):

- Each of these 5 experimental populations was initiated from a clone with an adaptive mutation in the RNA polymerase core enzyme that increases fitness under long-term stationary phase – but that is costly in rich media. (Avrani *et al.* 2020).
- One clone (2.1) had a normal mutation rate of $3.48 \cdot 10^{-9}$ per site. The other four clones were mutators, deficient in their mismatch repair, resulting in much higher mutation rates.
- Three of the clones (4.1, 4.5 and 4.9) had the same specific mutator allele, resulting in a mutation rate estimated to be 672-fold higher than that of the non-mutator clone.
- The remaining mutator clone (2.2). carried a different mutator allele resulting in a mutation rate 165-fold higher than that of the non-mutator.

These five experimental populations underwent daily dilutions into fresh media for 16 growth cycles, which corresponds to around 100 generations. During the serial dilution experiments, all five clones greatly improved the rate at which they grew exponentially in fresh media.

After 100 generations of evolution, 9-10 clones from each of the resulting populations were fully sequenced. This allowed us to examine whether the observed improvements in growth rate were achieved through compensation, reversion, or a combination of both. As can be seen, for the non-mutator populations, all 10 sequenced bacteria carried a compensatory mutation, while still maintaining their original costly mutation. While each clone carried a single compensatory mutation, four different compensatory mutations were observed within the population, indicating that this population adapted through a soft sweep (Hermisson and Pennings 2017). These results were quite similar to what we observed in our simulations, when $N\mu$ was set to 0.1 (Figure 4C) (Avrani *et al.* 2011). In the experiments with mutator strains, both compensatory and reversal mutations were observed (Figure 4F).

Literature cited

Avrani S, Katz S, Hershberg R. 2020. Ad aptations accumulated under prolonged resource exhaustion are highly transient. MSphere. 5. https://doi.org/10.1128/mSphere.00388-20.

Avrani S, Wurtzel O, Sharon I, Sorek R, Lindell D. 2011. Genomic island variability facilitates prochlorococcus –virus coexistence. Nature. 474:604–608. https://doi.org/10.1038/nature10172.

Hermisson J, Pennings PS. 2017. Soft sweeps and beyond: Understanding the patterns and probabilities of selection footprints under rapid adaptation. Methods in Ecology and Evolution. 8:700–716. https://doi.org/10.1111/2041-210X.12808.

Kimura M. 1957. Some problems of stochastic processes in genetics. The Annals of Mathematical Statistics. pp. 882–901.

Patwa Z, Wahl LM. 2008. The fixation probability of beneficial mutations. Journal of The Royal Society Interface. 5:1279–1289.
